# Menaquinone 4 Reduces Bone Loss in Ovariectomized Mice through Dual Regulation of Bone Remodeling

**DOI:** 10.3390/nu13082570

**Published:** 2021-07-27

**Authors:** Huakai Wang, Nan Zhang, Longxian Li, Pan Yang, Yongxi Ma

**Affiliations:** State Key Laboratory of Animal Nutrition, College of Animal Science and Technology, China Agricultural University, Beijing 100193, China; huakaiwhk@163.com (H.W.); S20203040629@cau.edu.cn (N.Z.); S20193040586@cau.edu.cn (L.L.); ypan23@163.com (P.Y.)

**Keywords:** menaquinone 4, ovariectomy, micro-CT, hematoxylin-eosin staining, mRNA expression

## Abstract

Epidemiologic studies showed that higher vitamin K (VK) consumption correlates with a reduced risk of osteoporosis, yet the dispute remains about whether VK is effective in improving bone mineral density (BMD). We sought to discover the anti-osteoporotic effect of menaquinone-4 (MK-4) and evaluate the expression of critical genes related to bone formation and bone resorption pathways in the body. Fifty female C57BL/6 mice (aged 13 weeks) were randomly arranged to a sham-operated group (SHAM, treated with corn oil) and four ovariectomized groups that were administered corn oil (OVX group), estradiol valerate (EV, 2 mg/kg body weight as the positive control), low or high doses of VK (LVK and HVK; 20 and 40 mg MK-4/kg body weight, respectively) by gavage every other day for 12 weeks. Body and uterine weight, serum biochemical indicators, bone microarchitecture, hematoxylin-eosin (HE) staining, and the mRNA expression of critical genes related to bone formation and bone resorption pathways were assessed. Either dose of MK-4 supplementation increased the alkaline phosphatase (ALP), decreased the undercarboxylated osteocalcin (ucOC) and tartrate-resistant acid phosphatase (TRACP, *p* < 0.05) levels, and presented higher BMD, percent bone volume (BV/TV), trabecular thickness (Tb.Th), and lower trabecular separation (Tb.Sp) and structure model index (SMI, *p* < 0.05) compared with the OVX group. Additionally, both doses of MK4 increased the mRNA expression of *Runx2* and *Bmp2* (*p* < 0.05), whereas the doses down-regulated *Pu.1* and *Nfatc1* (*p* < 0.05) mRNA expression, the high dose decreased *Osx* and *Tgfb* (*p* < 0.05) mRNA expression, and the low dose decreased *Mitd* and *Akt1* (*p* < 0.05) mRNA expression. These data show the dual regulatory effects of MK-4 on bone remodeling in ovariectomized mice: the promotion of bone anabolic activity and inhibition of osteoclast differentiation, which provides a novel idea for treating osteoporosis.

## 1. Introduction

Bone homeostasis is a dynamic balance between bone formation mediated by osteoblasts and resorption mediated by osteoclasts under physiological conditions [1]. The improvement of osteoblast activity or osteoclast activity will disrupt this dynamic balance and lead to abnormal bone metabolisms, such as osteopetrosis or osteoporosis [2]. Osteoporosis is a comprehensive bone disease caused by a variety of reasons, such as a decrease in BMD, an increase in bone fragility, and the destruction of the bone microstructure, etc. When the secretion of estrogen decreases or calcium is lacking, it will cause bone loss, low BMD, fragility, which lead to osteoporosis [3]. It threatens the health of middle-aged and the aged, especially postmenopausal women [4]. Osteoporosis is widely viewed as a global public health problem, and most of the clinical drugs used to treat osteoporosis are anti-resorption agents, such as bisphosphonates, calcitonin, estrogen, and its substitutes. The primary mechanism is to inhibit osteoclast activity and control the process of bone resorption [3]. Saito et al. [5] reported that anti-resorption agents only reduced the risk of non-vertebral fractures by 20%, and they cannot increase BMD. Teriparatide, abaloparatide, and romosozumab have been used to treat osteoporosis to stimulate the synthesis of new bone. Due to high prices and toxic side effects, their usage is restricted [6]. Therefore, in preventing and treating osteoporosis, there is an urgent need to seek alternative substances that can simultaneously increase the activity of osteoblasts and inhibit the osteoclastogenesis genes without toxic side effects.

VK is a general term for a class of 2-methyl-1, 4-naphthoquinones, and all their derivatives with clotting effects. Natural VK includes phylloquinone (K1) and menaquinone (K2), whose coagulation function was first discovered by Henrik Dam in 1929 [7]. VK_2_ has been a hot topic of scientific research in recent years, mainly focusing on its role in bone and cardiovascular function [8]; VK_2_ keeps the health of the body in many ways, including the prevention of cancer [9], the regulation of glucose and calcium metabolism [10], and the promotion of cell growth and proliferation [11]. Additionally, numerous clinical observations and animal experiments have shown that VK2 plays a key and dominant role in calcium deposition in bone during bone metabolism [12,13]. It is well known that excessive osteoclast activity and inadequate osteoblast activity can cause the development of osteoporosis [14]. VK_2_ can prevent osteoblast apoptosis, reduce osteoclast activity, and alleviate postmenopausal osteoporosis in ovariectomized rats [15,16]. However, some studies found insufficient evidence for the use of VK supplements to prevent bone loss, fracture, and increase BMD [17,18]. These findings are still controversial and deserve further investigation. Furthermore, the mechanism of VK_2_ in the improvement of osteoblast activity and the reduction of osteoclast activity remains unclear. In the present experiment, we systematically researched the effects of MK-4 on bone protection and the influences of the MK-4 on expressing critical genes related to bone formation and bone resorption pathways in ovariectomized mice.

## 2. Materials and Methods

This study was conducted in the Animal Nutrition Metabolism Laboratory at the Ministry of Agriculture and Rural Affairs Feed Efficacy and Safety Evaluation Center, which is located in China Agricultural University (Beijing, China). All animal procedures were approved by the Institutional Animal Care Committee of China Agricultural University (ICS: AW41501202—1—1).

### 2.1. Animals

Female C57BL/6 mice (aged 12 weeks) were purchased from the SiPeiFu Bioscience Co., Ltd. (Beijing, China). The animals were kept under normal cage conditions in a temperature-controlled room set at 25 °C, relative humidity of 65 ± 5% with 12-h light/12-h dark cycles, fed a standard diet, and allowed to acclimate for 7 d. Food and drinking water were freely supplied throughout the experiment. The diet composition is shown in Supplementary Table S1.

### 2.2. Experimental Protocols

After 7 d of adaptation, the sham operation or ovariectomy (including anesthesia) was carried out in line with the reference [19]. Three days after surgery, the animals were randomly divided into 5 groups: the corn oil control (SHAM, *n* = 10), ovariectomy with corn oil (OVX, *n* = 10), ovariectomy with estradiol valerate (EV, positive control, *n* = 10), ovariectomy with a low dose of MK-4 (LVK, *n* = 10), ovariectomy with a high dose of MK-4 (HVK, *n* = 10). MK-4 was dissolved in corn oil, and mice in the low- and high-dose groups received 20 or 40 mg/kg of body weight, respectively. These levels of VK_2_ in the present study were decided according to the previous study [18]. Mice in the EV group (EV was dissolved in corn oil) received 2 mg/kg of body weight based on previous research [19]. Mice in the SHAM and OVX groups received the same amount of corn oil, and all treatments were given by gavage every other day for 12 weeks. Mice were weighed weekly, and the dose adjusted according to their weight.

### 2.3. Sample Collection

After 12 weeks of treatment, blood was sampled from the orbital sinus. Serum was obtained by centrifugation at 3000× *g* for 10 min. The uterus, all tibia, and femur were also dissected. The serum, uterus, and tibia were stored at −80 °C. The femur was fixed in 4% paraformaldehyde and reserved at room temperature (BOSTER Biological Technology Co., Ltd., Wuhan, China).

### 2.4. Serum Biochemical Indicators

ELISA was conducted to determine mouse ucOC (CUSABIO Co., Ltd., Wuhan, China), ALP, and TRACP (Nanjing Jiancheng Bioengineering Institute, Nanjing, China), according to the manufacturers’ instructions.

### 2.5. Micro-CT Analysis

The left femur was used for micro-CT analysis as recommended by the American Society for Bone and Mineral Research [20]. High-resolution ex vivo micro-CT (Skyscan1174, Bruker Microtomograph, Kontich, Belgium) with a 12 μm voxel resolution, 50 kV voltage, 800 μA current, and a field size of 1304 × 1024 was used for image acquisition, and the scans were then integrated into 2D images. The bottom of the growth plate at the side of the femur knee joint was taken as the scanning reference line, and 83 continual sections were taken, that is, the area with a thickness of 1 mm was set as the 3D reconstruction region of interest, and bone morphology indexes were measured. For visualization, 3D images of the distal femur were reconstructed using the N-Recon software, and quantitative analyses of the morphometric parameters were conducted using the CT-AN software. The following trabecular morphometric indexes were analyzed: BMD, BV/TV, Tb.Th, Tb.Sp, trabecular number (Tb.N), and SMI. All analyses were conducted by individuals who were unaware of the specimen processing.

### 2.6. Hematoxylin-Eosin Staining

Right femurs were isolated and fixed in 4% paraformaldehyde, decalcified in 10% EDTA, and paraffin-embedded. The slice thickness was 5 μm and hematoxylin-eosin (HE) stained. For deparaffinization, the sections were soaked twice in xylene at 56 °C for 10 min; 100%, 90%, 80%, and 70% ethanol soaked for 5 min each; and water washed thrice for 5 min. All of the images were collected through microscopy (Olympus BX51, Olympus Corporation, Tokyo, Japan) to observe the arrangement of osteone, the boundary between the lacunae and bone matrix, and the bone cells of the compact bone.

### 2.7. Real-Time PCR for Gene Expression Analysis

Total RNA was extracted from the tibia using the EASY spin Plus Bone Tissue RNA Kit (Aidlab Biotechnologies Co., Ltd., Beijing, China). RNA quality and concentrations were measured using the NanoDrop 2000 (Thermo Fisher Scientific, Waltham, MA, USA). A cDNA with a total volume of 200 μL was generated by reverse transcription with 1 μg RNA. Real-time PCR was conducted with SYBR Premix EsTaq reagents using a StepOnePlus real-time PCR system (Applied Biosystems, Waltham, MA, USA). The relative mRNA expression was normalized to Actb. The primers for the real-time PCR are listed in Supplementary Table S2. The relative mRNA expression of the target gene was determined using the 2−ΔΔCt method [21].

### 2.8. Statistical Analysis

The UNIVARIATE procedure of SAS 9.2 (SAS Institute, Cary, NC, USA) was used to verify the normality of data. The significance of results was evaluated using a one-way ANOVA, followed by an appropriate Tukey’s multiple comparison test. *p* < 0.05 was considered statistically significant. The experimental data were expressed as the mean ± standard deviation (SD).

## 3. Results

### 3.1. Effect of MK-4 on Body and Uterine Weight in Ovariectomized Mice

**Figure 1:**
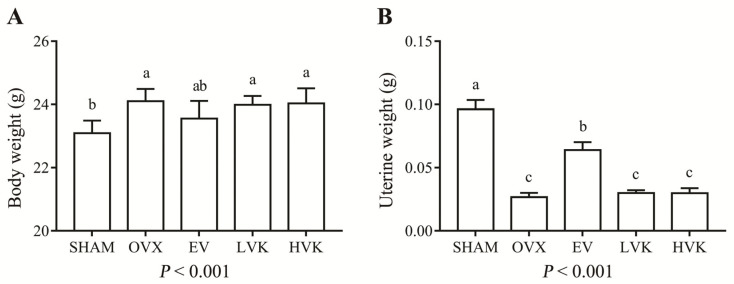
Body (**A**) and uterine (**B**) weights in mice that were sham operated or ovariectomized and untreated or administered EV, LVK, or HVK for 12 weeks. Values are mean ± SD, *n* = 8–10 per treatment. Labeled without a common letter means a significant difference, *p* < 0.05. EV, estradiol valerate; HVK; MK-4 40 mg/kg body weight group; LVK, MK-4 20 mg/kg body weight group; MK-4, menaquinone-4; OVX, ovariectomized group; SD, standard deviation.

At the beginning of the experiment, there were no significant differences among treatments in body weight. Body weights in the OVX group were higher than that of the SHAM group after 12 weeks (*p* < 0.05). However, treatment with EV or MK-4 did not affect body weights compared to those observed in the OVX group. Uterine weights of the ovariectomized mice were significantly decreased compared with those of mice in the SHAM group (*p* < 0.05; Figure 1B) and were not affected by the MK-4 treatment, but EV administration increased the uterine weights of the ovariectomized mice (*p* < 0.05).

### 3.2. Effect of MK-4 on Bone Metabolism-Related Serum Biochemical Indicators in Ovariectomized Mice

**Figure 2:**
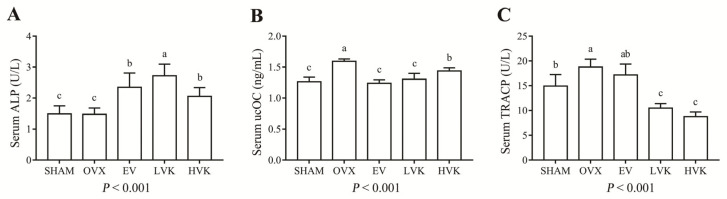
Serum ALP (**A**), ucOC (**B**), and TRACP (**C**) level in mice that were sham operated or ovariectomized and untreated or administered EV, LVK, or HVK for 12 wk. Values are mean ± SD, *n* = 8–10 per treatment. Labeled without a common letter means a significant difference, *p* < 0.05. ALP, alkaline phosphatase; EV, estradiol valerate; HVK; MK-4 40 mg/kg body weight group; LVK, MK-4 20 mg/kg body weight group; MK-4, menaquinone-4; OVX, ovariectomized group; SD, standard deviation; TRACP, tartrate-resistant acid phosphatase; ucOC, undercarboxylated osteocalcin.

Bone turnover markers are biochemical byproducts of bone formation or bone absorption [22]. There was no difference in the levels of ALP between the SHAM group and OVX group (Figure 2A), while treatment with EV or either dose of MK-4 significantly increased the ALP level in ovariectomized mice (*p* < 0.05). Serum levels of ucOC increased in the OVX group compared with the SHAM group (Figure 2B, *p* < 0.05), but EV or either dose of MK-4 treatment significantly reduced the levels of ucOC (*p* < 0.05) in the OVX group. The TRACP activity was also increased in the OVX group compared to the SHAM group (*p* < 0.05). The LVK and HVK groups showed lower levels of TRACP compared with the OVX group (*p* < 0.05), while the EV administration did not influence the serum levels of TRACP in ovariectomized mice.

### 3.3. Treatment of Ovariectomized C57BL/6 Mice with MK-4 Increases Bone Mass

**Figure 3:**
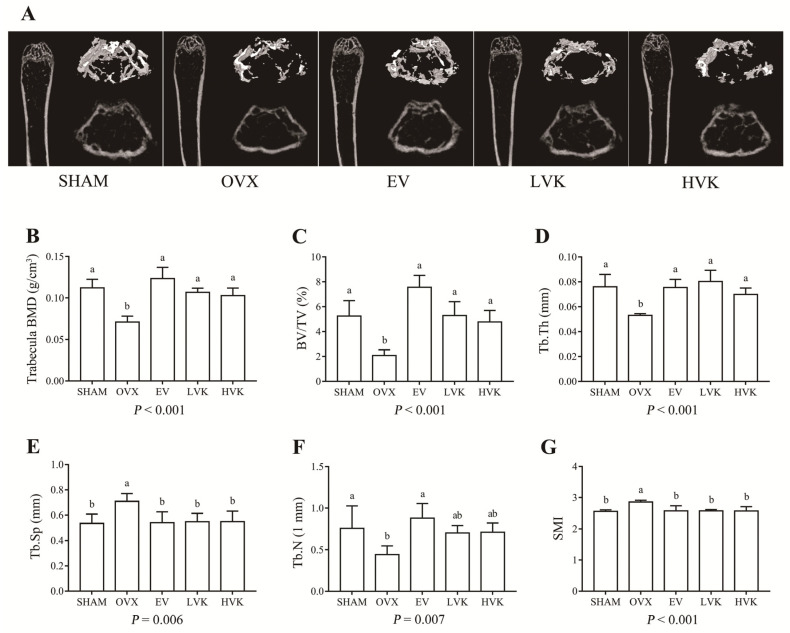
Bone microstructures in mice that were sham operated or ovariectomized and untreated or administered EV, LVK, or HVK for 12 weeks. Representative micro-CT images of the 3D reconstruction of distal femurs and cross sections (**A**). Quantitative analysis of the BMD, BV/TV, Tb.Th, Tb.Sp, Tb.N, SMI of trabecular bone by micro-CT (**B–G**). Values are mean ± SD, *n* = 5 per treatment. Labeled without a common letter means a significant difference, *p* < 0.05. BMD, Bone mineral density; BV/TV, percent bone volume; EV, estradiol valerate; HVK; MK-4 40 mg/kg body weight group; LVK, MK-4 20 mg/kg body weight group; MK-4, menaquinone-4; OVX, ovariectomized group; SD, standard deviation; BMD, bone mineral density; BV/TV, percent bone volume; Tb.Th, trabecular thickness; Tb.Sp, trabecular separation; Tb.N, trabecular number; SMI, structure model index.

The micro-CT images and the corresponding quantitative results are shown in Figure 3. In the OVX group, the levels of Tb.Sp and SMI (*p* < 0.05) were increased, while the trabecula BMD, BV/TV, Tb.Th, and Tb.N (*p* < 0.05) were decreased compared with the SHAM group. These results indicate bone mass was reduced in ovariectomized mice. Compared to the OVX group, treatment with EV and either dose of MK-4 increased the BMD, BV/TV, and Tb.Th (*p* < 0.05) and decreased the Tb.Sp and SMI (*p* < 0.05). Moreover, EV supplementation also increased the Tb.N (*p* < 0.05) compared with the OVX group. These data suggest that MK-4 can prevent ovariectomy-induced bone loss and effectively improve trabecula bone mass after a 12 weeks treatment.

### 3.4. Hematoxylin-Eosin Staining of Compact Bone in Ovariectomized Mice

**Figure 4:**
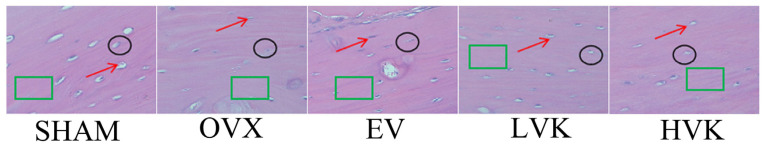
HE staining of compact bone in mice that were sham operated or ovariectomized and untreated or administered EV, LVK, or HVK for 12 weeks. *n* = 5 per treatment. The red arrows indicate bone cells, the black circles indicate the lacunae and bone matrix, and the green rectangles show osteone. EV, estradiol valerate; HVK; MK-4 40 mg/kg body weight group; LVK, MK-4 20 mg/kg body weight group; MK-4, menaquinone-4; OVX, ovariectomized group.

Tissue morphology of the femur was observed after HE staining (Figure 4). In the SHAM group, the cortical osteocytes of the femur were evenly distributed, the boundaries between lacunae and bone matrix were clear, and the arrangement of bone plates was relatively regular. However, cortical osteocytes of the femur were sparse, bone plates were disorganized, and the boundary between lacunae and bone matrix was fuzzy in the OVX group compared with those observed in the SHAM group. In contrast, treatment with EV or either dose of MK-4 effectively improved femur morphology.

### 3.5. Effect of MK-4 on Bone-Related Gene Expression in Ovariectomized Mice

**Figure 5:**
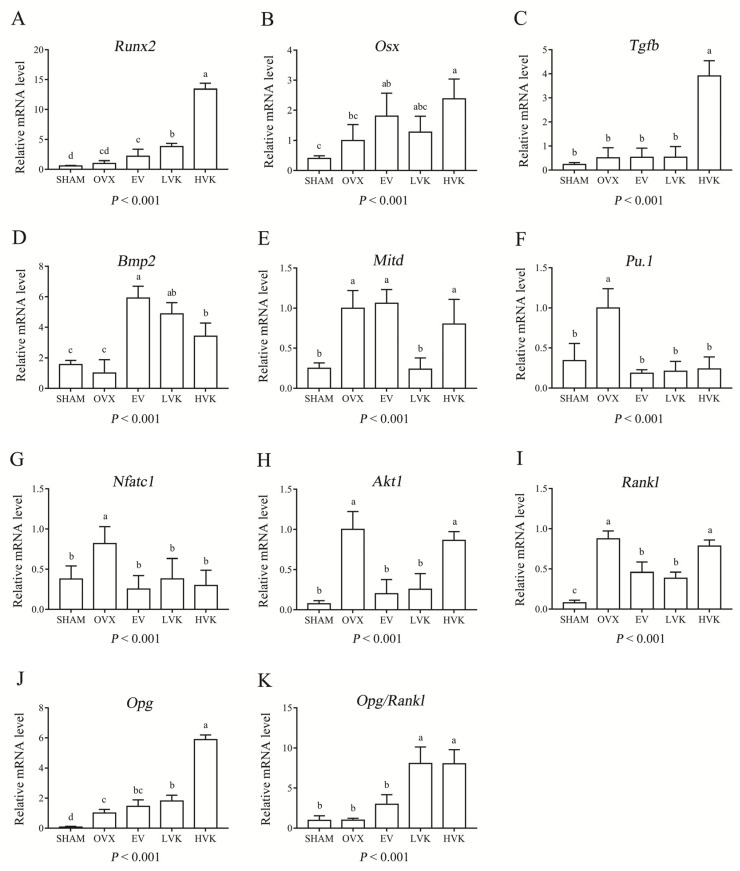
Genes related to bone formation and bone resorption in mice that were sham operated or ovariectomized and untreated or administered EV, LVK, or HVK for 12 weeks. The mRNA expression of *Runx2* (**A**), *Osx* (**B**), *Tgfb* (**C**), *Bmp2* (**D**), *Mitd* (**E**), *Pu.1* (**F**), *Nfatc1* (**G**), *Akt1* (**H**), *Rankl* (**I**), *Opg* (**J**), *Opg/Rankl* (**K**). Values are mean ± SD, *n* = 5–6 per treatment. Labeled without a common letter means a significant difference, *p* < 0.05. EV, estradiol valerate; HVK; MK-4 40 mg/kg body weight group; LVK, MK-4 20 mg/kg body weight group; MK-4, menaquinone-4; OVX, ovariectomized group; SD, standard deviation.

The real-time PCR data on the tibia showed there was no difference in the mRNA expression of *Runx2*, *Osx*, *Tgfb*, and *Bmp2* between the SHAM group and the OVX group (Figure 5A–D). However, treatment with both doses of MK-4 increased the mRNA expression of *Runx2* and *Bmp2* (*p* < 0.05), while the high dose increased *Osx* and *Tgfb* mRNA expression compared with the OVX group. Furthermore, treatment with EV increased the mRNA expression of *Bmp2* (*p* < 0.05) of the ovariectomized mice.

On the contrary, compared with the SHAM group, mRNA expression of *Mitd*, *Pu.1*, *Nfatc1*, and *Akt1* (*p* < 0.05) in the OVX group was significantly increased (Figure 5E–H). However, treatment with EV decreased the mRNA expression of *Mitd*, *Pu.1*, *Nfatc1*, and *Akt1* (*p* < 0.05) compared with the OVX group. Meanwhile, treatment with both doses of MK-4 decreased the mRNA expression of *Pu.1* and *Nfatc1* (*p* < 0.05), while the low dose decreased *Mitd* and *Akt1* mRNA expression compared with the OVX group. As shown in Figure 5I–K, mRNA expression of *Rankl* and *Opg* in the OVX group was increased compared with the SHAM group. However, there was no difference in the mRNA expression of *Opg*/*Rankl* between these two groups. Moreover, compared with the OVX group, mRNA expression of *Rankl* (*p* < 0.05) in EV and LVK groups was decreased, and mRNA expression of *Opg* (*p* < 0.05) in LVK and HVK groups was increased. Finally, treatment with MK-4 improved the *Opg*/*Rankl* ratio compared with the OVX group (*p* < 0.05).

## 4. Discussion

Osteoporosis leads to disability and is a significant disease burden to society that affects hundreds of millions of people worldwide, and the incidence is slowly increasing [23]. Given the limitations of osteoporosis treatments, safe and effective alternatives for preventing and treating osteoporosis that both promote osteogenic activity and inhibit osteoclast activity are needed. As a fat-soluble vitamin, VK_2_ may play a crucial role in bone metabolism and bone-related diseases. Clinical trials have pointed out that VK levels are associated with fracture and BMD, low dietary VK intake may increase the risk of fracture [24]. In addition, previous studies have shown that VK_2_ can improve intestinal Ca transport and prevent bone loss in vivo [18], improve serum bone metabolism-related indicators, and alleviate bone loss caused by osteoporosis through improvement in the bone microstructure [25]. This study used ovariectomized female mice as the animal model, which has been widely used in the study of estrogen deficiency-induced osteoporosis. We have characterized the bone phenotype of ovariectomized mice after treatment with MK-4. Our results show that MK-4 increased bone formation while decreasing bone resorption, which is associated with a higher expression of osteogenic-related pathway genes and a lower expression of osteoclast-related pathway genes.

It is well known that body-weight gain is a usual phenomenon observed in estrogen-deficient mice [26]. Additionally, ovariectomy can lead to a significant decrease in uterine weight, bone biology mechanics, and microstructure, partly due to estrogen deficiency [26]. Our results show that ovariectomy significantly increased the body weights of mice compared with the SHAM group. As previously described, estrogen secretion decreased in mice after ovariectomy, and bone mesenchymal stem cells (BMSCs) tend to differentiate into adipocytes, resulting in weight gain [27]. However, treatment with EV or MK-4 did not prevent weight gain in ovariectomized mice. Meanwhile, a lack of estrogen can lead to uterus atrophy in mice. Therefore, EV was used as an active treatment to prevent osteoporosis caused by estrogen reduction in our study. Our data indicate that treatment with EV significantly reduced the loss of uterine weight. However, MK-4 treatment did not appear to alleviate the reduction in uterine or body weights in ovariectomized mice, demonstrating that MK-4 may act through other mechanisms independent of estrogen supplementation to prevent bone loss.

It is established that the serum indicators of bone formation (ALP) and bone resorption (TRACP) are widely used to estimate bone remodeling [28]. Kim et al. [29] and Hwang et al. [30] found that estrogen deficiency decreased ALP and increased TRACP activity in ovariectomized mice. Osteocalcin is a critical matrix protein secreted by osteoblasts and plays a vital role in regulating bone calcium metabolism after carboxylation. Our data show that MK-4 treatment increased the ALP and ucOC while inhibiting the activity of TRACP. Taken together, MK-4 can improve bone metabolism-related serum indicators in ovariectomized mice through the promotion of osteoblast activity and inhibition of osteoclast activity.

The microstructure of trabecular bone is a critical factor in determining bone strength and the physiological function of bone [26]. The lack of estrogen in ovariectomized mice breaks the balance between osteoblasts and osteoclasts, and the osteoclast activity was enhanced, resulting in sparse bone trabeculae, an uneven distribution of bone cells, and the rapid loss of bone mass [19]. Micro-CT is a non-destructive 3D digital imaging technique used to study changes in bone structure and density during the incubation period in animal models of osteoporosis and osteoarthritis [31]. In the present study, BMD, BV/TV, Tb.Th, and Tb.N was significantly decreased, and Tb.Sp and SMI were increased in the ovariectomized mice after 12 wk. However, oral treatment of MK-4 reduced the damage to the bone microstructure through the above indicators. In addition, HE staining also reveal that MK-4 improved the microstructure of bone trabeculae, which was consistent with the micro-CT results. Therefore, these results indicate that MK-4 can improve bone micro-architectural properties in ovariectomized mice.

Bmps are members of the Tgfb superfamily and play critical roles in various biological processes [32]. Styrkarsdottir et al. [33] reported that the *Bmp2* gene is a strong candidate gene for bone formation and osteoblast differentiation and is related to a phenotype of low BMD and high-risk fractures. Tgfb superfamily members act through activating heterotetrameric complexes of type I and type II transmembrane Ser/Thr kinase receptors at the cell surface. These complexes transfuse intracellular signaling through a cascade of Smad complexes or mitogen-activated protein kinase (Mapk) reactions [34]. In this study, ovariectomy had no effect on the mRNA expression of *Bmp2* and *Tgfb*, but MK-4 treatment increased the mRNA expression of these two genes. Runx2 and Osx are two key transcription factors for promoting osteoblasts’ maturation and differentiation and are controlled by the canonical Tgfb/Bmp-Smad signaling pathway [35]. *Osx*-deficient mice only form cartilage, not bones, similar to the phenotype of *Runx2*-deficiency [36]. Ovariectomy did not influence the mRNA expression of *Runx2* and *Osx*, but MK-4 treatment increased the mRNA expression of both *Runx2* and *Osx* in ovariectomized mice. These data were in keeping with the findings of previous in vitro research [37]. In short, the above results indicate that ovariectomy mainly stimulates the enhancement of osteoclast activity leading to osteoporosis, and MK-4 treatment may work through activating the Tgfb/Bmp-2 signaling pathway.

Pu.1 and Mitd are two key transcription factors for osteoclast proliferation and early differentiation [38]. The mRNA expression of these two genes significantly increased in the OVX group compared with the SHAM group, and MK-4 treatment reversed this tendency. These results show that MK-4 can inhibit osteoclast activity and bone resorption. Akt1 is the major isoform of Akt (Akt1-3), and Akt signaling promotes osteoclast differentiation and survival [39]. Rankl and macrophage colony-stimulating factor (MCSF) can improve the survival, proliferation, and differentiation of osteoclasts partly by the activation of the Akt pathway [40]. The Rankl-Rank-Opg axis is the classic regulatory system of bone metabolism that regulates osteoclast differentiation [41]. The binding of Rankl to its receptor Rank leads to the rapid recruitment of intracellular signaling molecules, such as Mapk, Nfkb, Ap-1, Trafs, and Nfatc1, among which Nfkb is the most vital factor [42]. Furthermore, the Nfatc1 signal plays a pivotal role in the formation of osteoclasts, and inhibition of the Nfkb signal can inhibit the expression of *Nfatc1* [43]. Takatsuna et al. [44] demonstrated that *Nfkb* inhibitor suppresses Rankl induced osteoclastogenesis by down-regulating *Nfatc1* expression. In the present study, ovariectomy significantly up-regulated the mRNA expression of *Nfatc1* and *Akt1*, MK-4 treatment reversed the changes in the mRNA expression of these two genes in ovariectomized mice. Moreover, the *Opg* mRNA expression was increased after treatment with MK-4, but the *Rankl* mRNA expression was decreased. Overall, the ratio of *Opg*/*Rankl* was increased by treatment of MK-4. The results in this experiment were in keeping with previous studies reporting decreased mRNA expression of *Rankl* and the enhanced mRNA expression of *Opg* [45]. Briefly, these results demonstrate that MK-4 inhibited bone resorption possibly by down-regulating *Akt1* expression, up-regulating the ratio of *Opg*/*Rankl*, and down-regulating *Nfatc1* expression. One limitation of this study is that the mechanisms identified in vivo have not been further tested, and in addition, there is a lack of in vitro mechanism studies.

## 5. Conclusions

In conclusion, this study indicated that MK-4 increases BMD, restores the bone microarchitecture, and improves serum indicators of bone metabolism in ovariectomized mice by promoting bone formation and inhibiting bone resorption, possibly through the Tgfb/Bmp and Akt-Nfkb-Nfatc1 pathways. MK-4 may be a novel supplement for the prevention and treatment of osteoporosis.

## Data Availability

Not applicable.

## Supplementary Materials

The following are available online at https://www.mdpi.com/article/10.3390/nu13082570/s1, Table S1: The compositions of ingredients used in the experimental diets, Table S2: Primers for gene expression using real-time PCR.
