# Supplementary material for: Menaquinone 4 Reduces Bone Loss in Ovariectomized Mice through Dual Regulation of Bone Remodeling
Source: Nutrients. 2021 Jul 27;13(8):2570. doi: 10.3390/nu13082570 (PMC8398915; doi:10.3390/nu13082570)
Supplement: Supplementary file 1 [file nutrients-13-02570-s001.zip › nutrients-1303895-supplementary.pdf]

Table S1: The compositions of ingredients used in the experimental diets.

Items	Diets	Calculated nutrient, %	Value
Ingredients (g/kg)		Crude protein	18.00
Wheat feed flour	290.00	Ether extract	4.00
Bread flour	41.00	Crude Fiber	5.00
Extruded corn	408.40	Lysine	0.82
Fish meal	20.00	Methionine + cysteine	0.53
Soybean meal, 46%	160.60	Arginine	0.99
Soy oil	20.00	Tryptophan	0.19
Premix [a]	60.00	Histidine	0.55
		Phenylalanine tyrosine	1.10
		Threonine	0.65
		Leucine	1.44
		Isoleucine	0.70
		Valine	0.84

[a] Premix provided the following quantities per kilogram of the complete feed for mice: vitamin A (Retinyl Palmitate), 7000 IU; vitamin D (Cholecalciferol), 800 IU; vitamin E (Alpha tocopherol), 60 IU; vitamin K (Menadione sodium bisulfite), 3 mg; thiamin, 8 mg; riboflavin, 10 mg; vitamin B_6, 6 mg; vitamin B_{12}, 22 μg; biotin, 0.1 mg; niacin, 45 mg; pantothenate, 17 mg; folate, 4 mg; choline chloride, 1250 mg; Na, 2 g; K, 5 g; Mg, 2 g; Cu, 10 mg; Fe, 100 mg; Zn, 30 mg; Mn, 75 mg; I, 0.5 mg; Se, 0.15 mg; Ca, 15 g; P, 9 g.

Table S2. Primers for gene expression using real-time PCR.

Genes	-	sequence (5′-3′)	Size (bp)	NCBI Gene ID
Actb	Forward	GCAGGAGTACGATGAGTCCG	74	NM_007393.5
	Reverse	ACGCAGCTCAGTAACAGTCC		
Runx2	Forward	GCACCGACAGCCCCAACTT	72	NM_001271631.1
	Reverse	CCACGGGCAGGGTCTTGTT		
Osx	Forward	GATGGCGTCCTCTCTGCTT	144	XM_006520519.5
	Reverse	TATGGCTTCTTTGTGCCTCC		
Tgfb	Forward	AGAGACGTGGGGACTTCTTG	183	XM_036152883.1
	Reverse	GCTTTGGGGTGAAGTCTTCG		
Bmp2	Forward	CCGCTCCACAAACGAGAAAA	181	NM_007553.3
	Reverse	CAGCAAGGGGAAAAGGACAC		
Pu.1	Forward	ACCAACGTCCAATGCATGAC	201	NM_001378899.1
	Reverse	GCATCTGTTCCAGCTCCATG		
Mitd	Forward	CAAATGGCAAATACGTTACCCG	126	XM_036165908.1
	Reverse	CTCCCTTTTTATGTTGGGAAGGT		
Nfatc1	Forward	TGGGAGATGGAAGCAAAGACTGA	175	XM_036161029.1
	Reverse	CATTGGCAGGAAGGTACGTGAA		
Akt1	Forward	TTTTGTTTCTCGGATGCGCT	220	XM_006515415.2

	Reverse	TGGTCGCGTCAGTCCTTAAT		
Rankl	Forward	GAAACATCGGGAAGCGTACC	246	NM_011613.3
	Reverse	GTACGTCGCATCTTGATCCG		
Opg	Forward	CTGCACAGTGAGGAGGAAGA	164	XM_036159230.1
	Reverse	CACACTCACACACTCGGTTG		
